# LM-UNet: Lightweight Mamba-UNet Prostate MRI image segmentation network

**DOI:** 10.1371/journal.pone.0339719

**Published:** 2026-03-23

**Authors:** Kuncai Xu, Shuai Zhou, Yan Chen, Junhao Chen, Ning Zhang, Yilong Liao

**Affiliations:** 1 College of Intelligent Engineering, Guiyang Institute of Information Science and Technology, Guiyang, Guizhou, PR China; 2 Department of Radiology, Jiangmen Central Hospital, Jiangmen, Guangdong, PR China; University of Alberta, CANADA

## Abstract

Accurate segmentation of lesions in prostate magnetic resonance images (MRI) is important for assessing patient health and personalized treatment in the clinic. However, the traditional UNet segmentation network has low segmentation accuracy because of the fuzzy boundary and low contrast. Therefore, we propose a Lightweight Mamba-UNet (LM-UNet) prostate MRI image segmentation method. Initially, the encoder-decoder backbone structure consists of parallel vision mamba (PV-Mamba) and efficient multi-scale attention (EMA). The number of model parameters is reduced by constructing PV-Mamba while extracting the correlation between features over long distances. The EMA is then used to learn different spatial features in groups and construct cross-spatial information aggregation methods for richer feature aggregation. Subsequently, we construct the edge feature extraction (EFE) and the edge feature fusion (EFF) to achieve different levels of feature fusion in the encoder. Ultimately, we suggest a multi-stage and multi-level skip connections (MMSC) to achieve multi-level fusion between the encoder and decoder, there reducing semantic discrepancies between contextual features and improving segmentation accuracy. Experimental results demonstrate that on the PROMISE12 dataset, LM-UNet outperforms seven comparative segmentation methods in terms of parameter count, computational memory requirements, and precise segmentation of lesion margins.

## 1. Introduction

Prostate cancer is the most common malignant tumor in men and also one of the leading causes of male mortality. Approximately 1.1 million men worldwide are diagnosed with prostate cancer each year [1,2]. Currently, with the continuous increase in population aging, the incidence and mortality of prostate cancer in china are both gradually rising [3]. Early-stage prostate cancer can be effectively treated through surgery and can achieve good therapeutic outcomes. In contrast, advanced prostate cancer may metastasize, and once the cancer cells have spread, they are difficult to cure. Therefore, early screening, early detection, and early treatment of prostate cancer have significant clinical importance [4,5].

MRI has been widely used as a non-invasive imaging tool for the screening of prostate cancer. The clinician obtains the location, size, and shape of the region of interest in the prostate MRI image by segmenting it, so as to realize the diagnosis of the malignant degree of prostate cancer [6]. In this process, the diagnostic results are affected by the qualifications of clinicians, and the diagnostic consistency between different doctors is low [7,8]. Therefore, it is of great significance to develop an algorithm that can automatically segmentation prostate MRI images.

Deep learning techniques have shown good performance in medical image segmentation, mainly due to the use of convolutional neural network (CNN), a powerful modeling technique [9–11]. To address the challenge of low accuracy in medical image segmentation. Ronneberger et al. [12] proposed a fully convolutional UNet segmentation network. This architecture employs skip connections to integrate low-resolution features from the encoder downsampling process with high-resolution features generated through the decoder upsampling operations, effectively improving segmentation precision. However, due to the variability of lesions in medical images, multiple down-sampling of the UNet segmentation network will lead to the loss of detailed information such as edges. Song et al. [13] suggested a dual-branch framework comprising a global feature reconstruction and a local feature reconstruction, to preserve the global detail features of the input image. Yin et al. [14] developed a guided filtering module integrated after each downsampling and upsampling operation in the standard UNet architecture, establishing hierarchical feature guidance through inter-stage information propagation, achieve effective transmission of different features.

Furthermore, to resolve training instability caused by semantic discrepancies during the fusion of encoder-derived downsampled features and decoder-processed upsampled features. Asadi et al. [15] advanced a bidirectional convolutional long short-term memory that performs nonlinear integration of multi-scale representations by bidirectionally coupling hierarchical features from the encoding path with progressively refined outputs in the decoding pathway. To address edge information degradation in lesion segmentation. Zhu et al. [16] employed a boundary-weighted domain adaptive neural network that enhances boundary sensitivity during prediction, thereby achieving more precise extraction of boundary features in pathological images through adaptive feature recalibration. To precise delineation of anatomical structures in prostate lesion segmentation. Wang et al. [17] studied a boundary encoding network that learns discriminative representations of organ edge through multi-scale boundary-aware learning, by establishing dense contextual dependencies between boundary semantics and regional features to guide pixel-wise classification, achieving enhanced delineation accuracy of glandular contours in histopathology images.

Some researchers have improved the UNet and achieved good performance in prostate MRI image segmentation. However, these methods that use fully convolutional structures can only extract local features during the feature extraction process, lacking the ability to capture global features. Zhang et al. [18] employed a transfuse method with a parallel branch architecture, which can effectively capture inter-image dependencies and low-level spatial details, there improving the accuracy of traditional CNN for prostate segmentation. Hung et al. [19] designed a cross-slice attention using the transformer approach, which can be integrated with any skip-connection-based network architecture to achieve context information fusion. Pollastri et al. [20] investigated a transformer model based on long-distance self-supervised learning that integrates contextual information across various anatomical planes. Research has shown that segmentation methods based on the transformer only exhibit significant performance improvements when trained on large datasets [21,22]. Furthermore, the computational complexity of transformer-based segmentation methods is proportional to the square of the sequence length, which results in low efficiency when processing long sequences. This makes the network require a substantial amount of time and memory during both the training and inference.

To overcome the challenges of transformer massive parameter count, high training costs, and low efficiency in processing long sequences. Some scholars have focused their research on proposing numerous lightweight methods [23–26], which employs a selective mechanism to dynamically adjust state transition parameters based on input content, enabling focused detection of critical information such as lesion margins and fine structures. Simultaneously, leveraging its selective state space model, it achieves dynamic focusing on key lesion regions within medical images and long-range context modeling with linear computational complexity. Ensure global consistency and accuracy of the segmentation results, while significantly reducing the parameter and computational cost.

Overall,for the prostate MRI image segmentation, there may be some shortcomings using the existing UNet. For example, (1) the prostate MRI image data is small, while the UNet network constructed using full convolution has more parameters, and overfitting is easy to occur during model training. (2) During the encoding stage of the UNet network, multiple downsampling operations are performed, which leads to the loss of detailed edge information of the lesion. thus leading to unsmooth edge in the segmentation result. (3) The low resolution information in the direct encoding of the skip connection is fused with the high resolution information in the decoding, while there is a semantic difference between the two different resolution information itself, and the direct fusion will lose part of the spatial information, which leads to poor segmentation effect. Therefore, we propose the LM-UNet method for prostate MRI image segmentation, which can effectively segment lesions in prostate MRI images.

The contributions of this work are summarized as follows:

- We propose a LM-UNet method for prostate MRI image segmentation. LM-UNet consists of the parallel vision mamba (PV-Mamba), efficient multi-scale attention (EMA), edge feature extraction (EFE), edge feature fusion (EFF), and multi-stage and multi-level skip connections (MMSC) components. This method improves the accuracy of MRI image segmentation and captures correlation information between feature-length sequences while reducing network parameters and memory computation requirements.
- We construct the EFE and EFF modules. First, the EFE fuses the shallow texture details with the deep, abstract features from the encoder. Then, the EFF fuses each encoder output to enrich the diversity of the feature space.
- We put forward the MMSC to realize multi-stage and multi-level fusion of the fused features from the encoder with the decoder to reduce the semantic differences of the features between codecs and thus improve the segmentation accuracy.
- The lay out LM-UNet method is validated on the publicly available medical dataset PROMISE12 [27] and compared and analyzed with several classical segmentation methods, and the experimental results show that the segmentation method in this paper has the highest segmentation performance.

## 2. Methods

**Fig 1:**
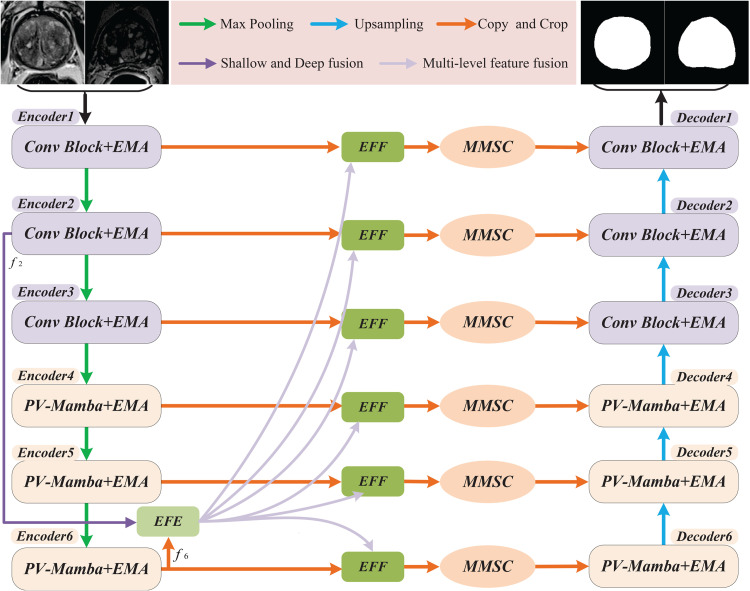
LM-UNet Architecture. The encoder and decoder of LM-UNet consist of PV-Mamba+EMA, with skip connections formed by EFF and MMSC. EFE is employed to fuse edge features from different levels of encoder layers $f_{2}$ and $f_{6}$.

In this section, we present a method for segmenting prostate MRI images using an LM-UNet, based on the encoding and decoding ideas of the UNet network. As demonstrated in Fig 1, the LM-UNet is primarily made up of an encoder, a decoder, and a skip connection.

### 2.1. Method overview

To achieve efficiently segment of prostate MRI images, we have designed a novel LM-UNet prostate MRI image segmentation network based on the encoding and decoding ideas of UNet network, as observed in Fig 1. LM-UNet is mainly composed of encoder, decoder, and MMSC. The encoder-decoder are composed of PV-Mamba, EMA, and EFE. As a selective state space model with linear computational complexity, PV-Mamba can effectively capture the relevant information between long sequences, while reducing network parameters and memory computing requirements [28]. EMA uses cross-spatial learning to process feature aggregation in parallel, which avoids the problem of over-fitting caused by too deep network. EFE embeds low-level edge features into high-level semantic features, effectively realizing the fusion of shallow features and deep features. MMSC performs multi-stage and multi-level fusion output of the fusion features from the encoder and is connected to the decoder to reduce the semantic difference between the contexts and enhance the expression ability of the features, thereby improving the segmentation accuracy.

### 2.2. Encoder-Decoder

The encoder and decoder comprise of PV-Mamba, EMA, and EFE components. The functions of each component are as follows.

#### 2.2.1. PV-Mamba.

**Fig 2:**
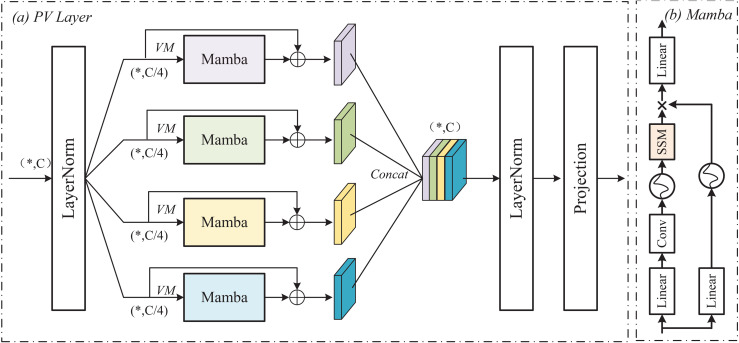
Parallel PV-Mamba Architecture.

To reduce the computational complexity of the network and capture the relevant information between long sequences, we introduce the PV-Mamba in layers 4–6 of LM-UNet. As indicated in Fig 2, PV-Mamba is a selective state space model with linear computational complexity that excels at handling long sequence modeling. PV-Mamba through the global receptive field and dynamic weighting mechanism, it effectively alleviates the limitations of convolutional neural networks in modeling and realizes the long-distance modeling ability with transformer. The detailed steps of PV-Mamba operation are as follows.

(1) Apply LayerNorm processing to the feature map *X* output from the third-layer encoder to reduce internal covariate shifts and enhance stability. The LN feature map is divided into four sub-feature maps $Y_{1}^{C/4},Y_{2}^{C/4},Y_{3}^{C/4}$ and $Y_{4}^{C/4}$, each sub-feature map has one-fourth the number of channels of the original feature map, i.e., *C*/4, with the aim of reducing the computational complexity of each sub-feature map.
(2) Each sub-feature map is fed into the Mamba for processing. The PV-Mamba optimizes feature representations by introducing dynamic weights and a global receptive field, enabling the to capture richer feature information [29]. The output processed by PV-Mamba is added to the residual of the original sub-feature map to obtain feature $PV\_Y_{i}^{C/4}$. Specifically, $PV\_Y_{i}^{C/4}$ is formed by summing the feature obtained by scaling the original sub-feature map by adjustment factor *α* and the feature output by PV-Mamba. This helps mitigate the vanishing gradient problem in deep networks and allows the model to flexibly adjust the weighting between original features and learned features.
(3) By concat operation the information from each sub-feature map, the feature map $X_{out}$ is obtained.
(4) The concatenated feature map $X_{out}$ is passed through an LN layer to ensure consistency in feature distribution. A projection operation is then applied to adjust the feature map dimensionality, ensuring it meets the input requirements of subsequent network layers. The calculation formula for PV-Mamba is as follows:

$$Y_{1}^{C/4},Y_{2}^{C/4},Y_{3}^{C/4},Y_{4}^{C/4}=\text{Sp}[LN(X^{C})]$$ (1)

$$PV\_Y_{i}^{C/4}=Mamba(Y_{i}^{C/4})+\alpha \cdot Y_{i}^{C/4},\;i=1,2,3,4$$ (2)

$$X_{out}=\text{Cat}(PV\_Y_{1}^{C/4},PV\_Y_{2}^{C/4},PV\_Y_{3}^{C/4},PV\_Y_{4}^{C/4})$$ (3)

$$\text{Out}=\text{Pro}[LN(X_{out})]$$ (4)

where LN is the LayerNorm, Sp  means the split operation, *α* indicates the adjustment factor for the residual addition, *Cat* represents the concat operation, and Pro  denotes the projection operation. From Eqs (1)–(4), it can be seen that the use of PV-Mamba processing features ensures that the total number of channels processed remains constant and the maximization parameter is reduced while maintaining high accuracy.

#### 2.2.2. Efficient multi-scale attention.

**Fig 3:**
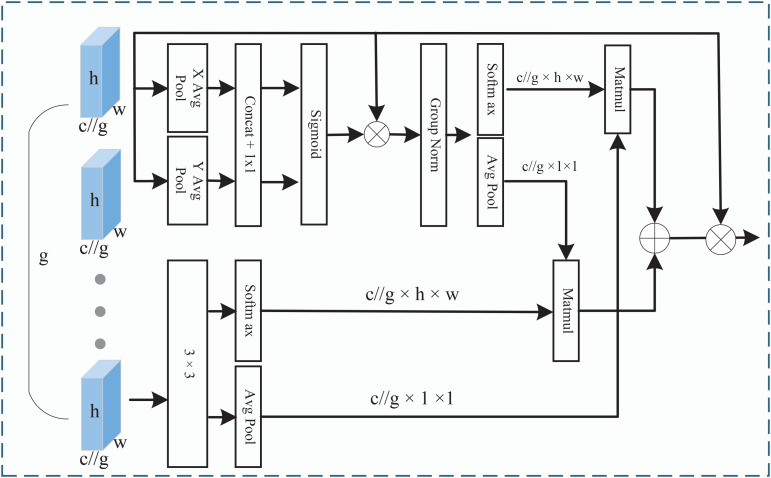
Efficient Multi-scale Attention.

To preserve complete channel information while effectively learning features, we introduced an EMA after each encoder backend. This alleviates the information bottleneck caused by increased network depth while enhancing cross-spatial feature interaction and aggregation capabilities [30]. As demonstrated in Fig 3, Firstly, EMA partitions the input features into multiple groups along the channel dimension to reduce computational complexity while preserving complete channel information. Subsequently, a parallel subnetwork was constructed to extract spatial and channel-wise attention weights for different groups, thereby capturing cross-regional semantic dependencies. Ultimately, a cross-spatial information fusion mechanism was designed. By leveraging multi-scale feature interaction and weighted aggregation, it dynamically integrates discriminative local features from different groups with global contextual information. This approach significantly enhances the model representational capacity while preserving feature resolution. The operation is as follows.

**Feature Grouping**: The EMA uniformly partitions the input feature map $X\in {\mathbb{R}}^{C\times H\times W}$ along the channel dimension, dividing it into *g* mutually exclusive sub-feature groups $\left[X_{0},X_{1},\ldots ,X_{g-1}\right]$, where each sub-feature has a dimension of $X_{i}\in {\mathbb{R}}^{C/g\times H\times W}$. This *g*rouping strategy achieves structural decoupling of features while avoiding channel compression and preserving the integrity of original feature information.

**Parallel Sub-Network**: EMA extracts attention weights for group features through three functionally complementary parallel paths. Two paths perform average pooling operations along the height and width of the feature map, respectively, yielding two feature maps. Then, the features are refined using concat and 1×1 convolutions, and Sigmoid activation is applied to generate attention weights. Next, multiply the generated attention weights element-wise with the original input feature map to weight the input feature map. Second, the weighted feature maps undergo group normalization. The group-normalized feature maps are then processed using Softmax to generate normalized attention weights, and use Matmul to weight the attention weights with feature maps. The third approach employs 3×3 convolutions, focusing on capturing multi-scale local spatial features to enhance feature distinguishability. Finally, the outputs of all paths are multiplied element-wise to generate the final output feature map. This parallel processing architecture integrates global context with local details, there providing more robust attention weights for subsequent feature aggregation.

**Cross-Space Learning**: During the cross-spatial learning stage, EMA can fuse features from different spatial dimensions. For the outputs of two 1×1 convolutions, a two-dimensional global average pooling operation is applied to encode global spatial information, there compressing the feature map of each channel into features with a global receptive field. Meanwhile, the output features of the 3×3 branch undergo dimensionality transformation to be reshaped into a dimension structure matching that of the former. Ultimately, the output features of EMA encompass both captured remote spatial dependencies and extracted multi-scale local details, enabling cross-channel fusion.

#### 2.2.3. Edge feature extraction module.

**Fig 4:**
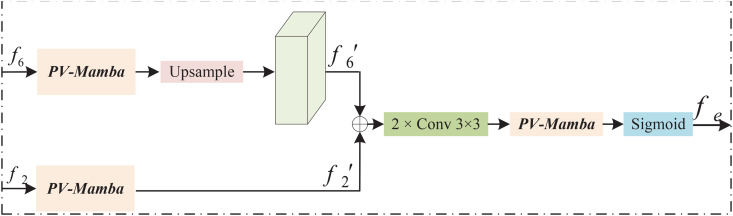
The Edge Feature Extraction Architecture.

A good edge prior can improve the accuracy of segmentation results. Although low-level features contain rich edge details [31], they also introduce numerous edges from regions of no interest. To enhance features along edges of interest, an edge-aware module is proposed to extract edge information from prostate MRI images. As shown in Fig 4, EFE combines the low-level feature $f_{2}$ and the high-level feature $f_{6}$ within the encoder. $f_{2}$ and $f_{6}$ are processed by PV-Mamba respectively, followed by integrating feature $f_{2}^{\prime }$ and the upsampled $f_{6}^{\prime }$ through concatenation. Finally, feature enhancement is performed using two 3×3 convolutional layers, and edge features $f_{e}$ are obtained by activating them with PV-Mamba and Sigmoid functions.

### 2.3. Multi-stage multi-level skip connections

MMSC include EFF, multi-stage channel attention (MCA), and multi-stage and multi-scale spatial attention (MMSA) components. The EFF fuses the shallow information with each encoder output and embeds the edge information into each encoder to provide the decoder with a priori knowledge. The MCA realizes multi-stage and multi-scale information enhancement in the encoder to efficiently extract target information of different sizes in prostate MRI. The MMSA module enhances the spatial dimension features thereby enhancing the feature representation.

#### 2.3.1. Edge feature fusion.

**Fig 5:**
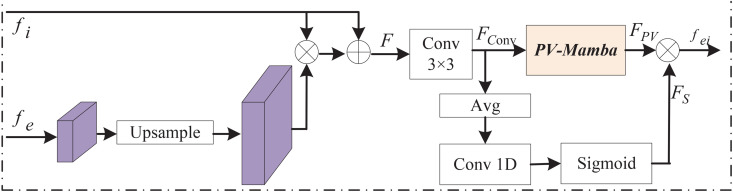
The Edge Feature Fusion Architecture.

To enrich the diversity of the feature space, the EFF injects the EFE output into the feature representation to improve the representation of the lesion structure semantics. As shown in Fig 5, given the input feature $f_{2}-f_{6}$ and edge feature $f_{e}$. The features from $f_{i}$ and the features after $f_{e}$ upsampling are multiplied element by element and connected with $f_{i}$ residual. Then 3×3 convolution was used to learn the fused features, and the fused features were fed into the PV-Mamba to construct long-distance dependencies to enhance the correlation between the features. To enhance the fusion feature representation, the fusion features are processed using average pooling (Avg), and then the corresponding channel attention weights are obtained by 1D convolution and *Sigmoid* function and multiplied with the output of PV-Mamba to obtain the fusion of each encoder with the edge features as $f_{ei}$. The EFF is calculated as follows.


$$F=f_{i}+f_{i}\times U(f_{e})$$ (5)

$$F_{\text{conv}}={\text{conv}}_{3\times 3}(F)$$ (6)

$$F_{\text{PV}}=PV(F_{\text{conv}})$$ (7)

$$F_{s}=\sigma (\text{conv1D}(Avg(F_{\text{conv}})))$$ (8)

$$f_{ei}=F_{\text{PV}}\times F_{S}$$ (9)

where $f_{i}$ is the output of the *i* encoder, $f_{e}$ denotes the edge feature, and *PV* indicates the Mamba operation, *σ* is a *Sigmoid* function, and ⊗ means the element-wise multiplication.

#### 2.3.2. Multi-stage channel attention.

**Fig 6:**
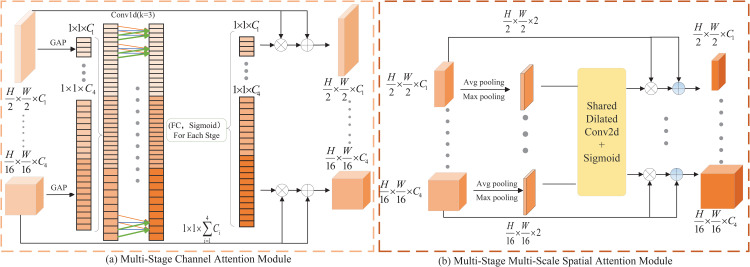
Multi-stage Multi-level Skip Connections.

Obtaining multi-stage and multi-scale information in the encoder is crucial for segmenting targets of different sizes in prostate MRI. The fusion of multi-stage and multi-scale information has been proven to be key to improving segmentation performance [25,32]. Therefore, a MCA for channel level is proposed to generate channel attention map, which is generated by connecting features of different stages on the channel axis. As shown in Fig 6 (a), MCA fuses multi-stage and multi-scale information into local information fusion (1D convolution operation) and global information fusion(different fully connected layers in each stage) to provide more informative attention feature maps. To better integrate multi-stage and multi-scale information from the encoder. The specific operation of MCA is as follows.


$$t_{i}^{\prime }=GAP\left(t_{i}\right)$$ (10)

$$T=\text{Concat}\left(t_{1}^{\prime },t_{2}^{\prime },\ldots ,t_{S-1}^{\prime }\right)$$ (11)

$$T^{\prime }=\mathrm{Conv}1D(T)$$ (12)

$$At_{i}=\sigma \left(FC_{i}\left(T^{\prime }\right)\right)$$ (13)

$$Out_{i}=t_{i}+t_{i}⊗Att_{i}$$ (14)

where *GAP* stands for global average pooling, $t_{i}$ represents the feature maps of different stages obtained from the encoder, *Concat* is the join operation on the channel dimension, *Conv1D* indicates one-dimensional convolution operation, $FC_{i}$ is the fully connected layer of stage *i*, *σ* is a *Sigmoid* function, and ⊗ is the element-wise multiplication.

#### 2.3.3. Multi-scale multi-spatial attention.

The MMSA is proposed to generate spatial attention maps for each stage to integrate multi-scale information on the spatial dimension [33]. The MMSA is shown in Fig 6 (b), To begin with, the average pooling and maximum pooling operations are used to map the features of each stage. Moreover, the dilated convolution (dilation rate is 3, kernel size is 7) and *Sigmoid* are used for normalization to generate spatial attention at each stage. Lastly, the generated spatial attention map is multiplied by the original feature map element by element to enhance the features of important regions and suppress the features of unimportant regions. The enhanced feature map is added to the original feature map through residual connection, and the spatial attention information is enhanced while retaining the original features. MMSA operation is as follows.


$$F_{\text{avg}}=\text{AvgPool}(F_{i})$$ (15)

$$F_{\text{max}}=\text{MaxPool}(F_{i})$$ (16)

$$F_{\text{cat}}=\text{Concat}(F_{\text{avg}},F_{\text{max}})$$ (17)

$$F_{\text{att}}=\sigma (\text{DilatedConv1D}(F_{\text{cat}}))$$ (18)

$$F_{\text{out}}=F_{i}+F_{i}⊗F_{\text{att}}$$ (19)

where $F_{i}$ is the feature map of stage *i*, *AvgPool* represents average pooling, *MaxPool* indicates MaxPool, *Concat* denotes a connection operation, *σ* is a *Sigmoid* function, and ⊗ means the element-wise multiplication.

### 2.4. Loss function

In the image segmentation, the goal is to enable the model to distinguish different regions. The cross entropy loss function helps the model learn how to distinguish different categories more accurately by minimizing the difference between the predicted probability distribution and the real distribution. Therefore, this paper uses cross entropy loss constraint model training, which is defined as follows:


$$l_{\text{bce}}=-\frac{1}{n}\sum \left[y\ln(\text{sigmoid}(\hat{y}))(1-y)\ln(1-\text{sigmoid}(\hat{y}))\right]$$ (20)

where *y* is the true label and $\hat{y}$ means the prediction. In medical image segmentation, there is a need to face the difficult problem of unbalanced data samples, if the cross-entropy loss function alone is used to constrain the model training without considering the category problem, dice similarity coefficient is used to measure the degree of overlap between predicted segmentation and real segmentation. Combining the cross-entropy loss function and the dice loss function can simultaneously optimize the classification accuracy of the model and the consistency of the segmentation, which is especially important for medical images that require accurate segmentation [34]. The definition is as follows.


$$Dice=\frac{2\sum_{i}^{N}p_{i}g_{i}}{\sum_{i}^{N}p_{i}^{2}+\sum_{i}^{N}g_{i}^{2}}$$ (21)

where $p_{i},g_{i}$ is the dot product summation operation between the predicted segmentation results and the labels, and the dice loss expression is:


$$l_{Dice}=1-\frac{2\sum_{i}^{N}p_{i}g_{i}}{\sum_{i}^{N}p_{i}^{2}+\sum_{i}^{N}g_{i}^{2}}$$ (22)

In this paper, a combined loss function is used for constrained model training, combining the two functions to optimize the loss calculation of the network model locally and holistically to improve the network model segmentation accuracy. The definition of combined loss is as follows:


$$l=l_{BCE}+l_{Dice}$$ (23)

where $l_{BCE}$ is the cross-entropy loss function and $l_{Dice}$ denotes the dice loss function.

### 2.5. Architecture comparison

Table 1 illustrates the differences between LM-UN and UVM-UNet. LM-UNet incorporates EMA and EFE into the encoder to extract edge features at distinct hierarchical levels. The skip connections encompass EFF, MCA, and MMSA, enhancing cross-spatial interactions and multi-level feature fusion. This precisely captures prostate edges, thereby improving segmentation performance.

**Table 1:** Detailed Comparison of LM-UNet and UVM-UNet.

Architecture	LM-UNet	UVM-UNet
**Encoder**	**ConvBlock**:Extract low-level structures such as local edges and textures, then progressively abstract them into higher-level semantic patterns.	**Conv Block**:Extract low-level structures such as local edges and textures, then progressively abstract them into higher-level semantic patterns.
	**EMA**:Cross-spatial parallel interaction alleviates information bottlenecks and enhances representation.	**PV-Mamba**:Channel-wise linear scanning enables long-range dependency modeling with a global receptive field.
	**PV-Mamba**:Channel-wise linear scanning enables long-range dependency modeling with a global receptive field.	
	**EFE**: Extract prostate edge priors at different levels, suppress irrelevant edges, and enhance target boundary features.	
**Decoder**	**Conv Block**:Extract low-level structures such as local edges and textures, then progressively abstract them into higher-level semantic patterns.	**Conv Block**:Extract low-level structures such as local edges and textures, then progressively abstract them into higher-level semantic patterns.
	**EMA**:Cross-spatial parallel interaction alleviates information bottlenecks and enhances representation.	
	**PV-Mamba**:Channel-wise linear scanning enables long-range dependency modeling with a global receptive field.	**PV-Mamba**:Channel-wise linear scanning enables long-range dependency modeling with a global receptive field.
**Skip Connections**	**EFF**:Fusing features from different levels within the encoder explicitly enhances the semantic characteristics of lesion edges.	**SAB**:Extract spatial features
	**MCA**:Generate channel attention weights to achieve cross-scale channel enhancement of effective features.	**CAB**:Extract channel features
	**MMSA**:Generate spatial attention weighting for multi-scale spatial feature enhancement.	

## 3. Experimental results and analysis

### 3.1. Datasets

#### 3.1.1. Data acquisition.

In this study, we validated all segmentation methods using the PROMISE12 dataset, a widely recognized public medical image dataset. In prostate MRI data acquisition, transverse T2-weighted MRI was selected as the primary imaging modality due to its significant advantages in anatomical detail. This imaging technique is widely adopted in clinical diagnosis and research for its high soft tissue contrast and ability to clearly display the prostate and its surrounding structures. It provides detailed views of the prostate internal architecture, including differentiation between the prostatic capsule, central gland, and peripheral gland. PROMISE12 data from 4 centers, including university college london (UCL), the beth israel deaconess medical center (BIDMC), haukeland university hospital (HK) and the radboud university nijmegen medical centre (RUNMC) [27]. Detailed information regarding data collection is provided in Table 2.

**Table 2:** Detailed Information on Data Collection for Each Center.

Center	Field Strength	Endorectal coil	Resolution	Manufacturer
			(in-plane/through-plane mm)	
UCL	1.5 and 3T	No	0.325–0.625/3–3.6	Siemens
BIDMC	3T	No	0.25/2.2–3	GE
HK	1.5T	Yes	0.625/3.6	Siemens
RUNMC	3T	Yes	0.5–0.75/3.6–4.0	Siemens

#### 3.1.2. Data description.

To validate the effectiveness of LM-UNet, the experimental data set uses the PROMISE12 data set proposed by the medical image computing and computer assisted intervention society (MICCAI) at the prostate MRI image segmentation challenge held in 2012. The training set consists of 1,473 images, while the test set consists 473 images. Moreover, tissue sections from the same patient will not appear in both the training and testing sets simultaneously.

#### 3.1.3. Data preprocessing.

We preprocessed the PROMISE12 dataset to improve image quality and enhance feature information. The preprocessing steps are as follows:

**Step 1**: Convert NIFTI data to PNG format. To enhance image clarity and reduce noise interference, we employ histogram equalization to adjust brightness and contrast, thereby boosting overall image contrast and achieving a more uniform brightness distribution.

**Step 2**: After clinicians locate the mask with the largest diameter for each patient, the center of the mask is determined using OpenCV. A bounding rectangle is then drawn around the lesion area with the center coordinates as the origin, ensuring the lesion is fully enclosed. Then, expand each of the top, bottom, left, and right edge of the rectangle by 5 pixels to ensure that the edge near the lesion area are fully encompassed.

**Step 3**: Apply the expanded rectangle coordinates to smaller mask images of other lesion areas in the same patient, then crop the corresponding regions to obtain the entire lesion area. Overlay the obtained mask region onto the corresponding MRI image to isolate the relevant lesion area.

**Step 4**: To meet the input requirements of deep neural network models, the lesion area was normalized to a size of 224×224 pixels, all data are three-channel images.

### 3.2. Implementation details

In this experiment, all methods were trained on a Linux 6.1.85 system equipped with an Nvidia L4 graphics card, using the PyTorch 1.13 + cu117 deep learning framework. During training, all models employ identical configurations. The batch size is 16, and the training for 100 epochs. The AdamW optimizer is uniformly applied,with lr=0.001, betas=(0.9,0.999), $eps=e^{-8}$ and $weight\_decay=1\times 10^{-2}$. The input channel sizes of the encoder are 3, 8, 16, 24, 32, and 48, respectively. The corresponding output channels are 8, 16, 24, 32, 48, and 64, respectively. The input and output dimensions of the encoder are shown in Table 3. The decoder output corresponds to the encoder output. The output sequence from Encoder 3 converts the two-dimensional feature map into a one-dimensional sequence via grid scanning. After transposition, it becomes [B, 3,136, 24] and is input into Mamba. The state dimension is 16, with a minimum discretization step size of 0.001 and a maximum discretization step size of 0.1. To evaluate the model generalization capability, we employed 5-fold cross-validation during the LM-UNet training process. This method was used to select the optimal model and hyperparameters, there enhancing the model reliability and performance.

**Table 3:** LM-UNet encoder input and output dimensions.

Encoder	Input Dimension [*B,C,H,W*]	Operation	Output Dimension [*B,C,H,W*]	Sequence length *L*
Encoder 1	[B,3,224,224]	Initial Conv	[B,8,224,224]	50,176
Encoder 2	[B,8,224,224]	Downsampling	[B,16,112,112]	12,544
Encoder 3	[B,16,112,112]	Downsampling	[B,24,56,56]	3,136
Encoder 4	[B,24,56,56]	Downsampling	[B,32,28,28]	784
Encoder 5	[B,32,28,28]	Downsampling	[B,48,14,14]	196
Encoder 6	[B,48,14,14]	Downsampling	[B,64,7,7]	49

### 3.3. Validation metrics

To evaluate the performance of different segmentation method, we employed the following metrics: Dice Similarity Coefficient (DSC), Intersection over Union (IoU), Accuracy, Specificity, Sensitivity, HD95, Precision, and Average Symmetric Surface Distance (ASSD) to objectively, and quantitatively evaluate the segmentation algorithm.

### 3.4. Experimental result

To further validate the effectiveness of LM-UNet, we conducted comparative analyses with segmentation methods, including UNet [12], ResUNet [35], PraNet [36], TransUNet [37], SwinUNet [38], VM-UNet [39], and UVM-UNet [28].

The evaluation metrics for the different segmentation methods are shown in Table 4. The LM-UNet had the highest DSC, IoU, Accuracy, HD95, Precision and ASSD which reached 92.75, 86.81, 96.57, 4.37, 92.95, and 3.95, respectively. The Specificity and Sensitivity were next best with 97.74 and 93.30. The evaluation results show that LM-UNet has the best segmentation performance on PROMISE12 dataset.

**Table 4:** Performance comparison of different methods.

Method	DSC	IoU	Accuracy	Specificity	Sensitivity	HD95	Precision	ASSD
UNet	90.86 ± 6.39	83.77 ± 8.90	95.66 ± 1.83	**97.90 ± 2.07**	90.25 ± 7.66	5.88 ± 4.38	92.61 ± 9.71	4.94 ± 2.20
ResUNet	87.52 ± 13.52	79.78 ± 16.70	94.75 ± 3.28	96.05 ± 3.24	89.58 ± 14.82	8.71 ± 8.23	87.32 ± 13.77	5.95 ± 4.01
PraNet	91.63 ± 5.44	84.96 ± 8.33	95.98 ± 1.81	97.69 ± 2.23	91.95 ± 6.69	5.34 ± 4.10	92.36 ± 9.19	4.61 ± 2.23
TransUNet	91.33 ± 6.92	84.67 ± 9.95	95.99 ± 1.93	97.24 ± 2.40	92.52 ± 9.05	5.41 ± 4.52	91.53 ± 9.13	4.66 ± 2.66
SwinUNet	87.63 ± 8.94	78.84 ± 11.09	93.68 ± 3.45	93.02 ± 3.97	**94.93 ± 8.68**	8.79 ± 6.36	82.58 ± 11.62	6.14 ± 2.82
VM-UNet	90.72 ± 5.69	83.46 ± 8.79	95.55 ± 1.99	97.27 ± 2.31	91.19 ± 8.29	6.16 ± 4.31	91.47 ± 8.99	5.03 ± 2.34
UVM-UNet	91.33 ± 5.45	84.45 ± 8.26	95.82 ± 1.72	97.35 ± 2.23	92.20 ± 6.86	5.50 ± 3.70	91.57 ± 9.22	4.81 ± 2.21
**LM-UNet**	**92.75 ± 4.65**	**86.81 ± 7.44**	**96.57 ± 1.46**	97.74 ± 1.87	93.30 ± 6.29	**4.37 ± 3.28**	**92.95 ± 7.52**	**3.95 ± 1.86**

**Fig 7:**
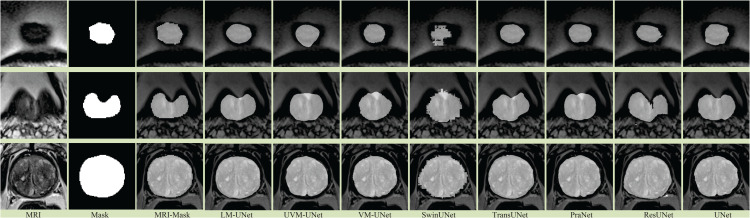
Segmentation Results of Different Segmentation Methods on PROMISE12 Dataset.

The visualization of prostate segmentation results by each segmentation method is shown in Fig 7. The first and second columns are prostate MRI images and corresponding mask. See Fig 7, it can be seen that when segmenting prostate MRI images, the ResUNet has the worst segmentation results, and the segmentation have more missegmentation, and even the lesion area is not segmented. The reason may be that the data is less, resulting in model training overfitting. The segmentation methods of UNet and PraNet can segment the general area of the lesion, but there are also some under-segmented areas, and the segmentation edge is blurred, with more burrs. The main reason is that the segmentation method based on CNN can only extract the local features of the image and lose the global information of the image. In addition, in the process of skip connection, the results of multiple down-sampling in the encoder are directly fused with the decoder, and there is a large semantic gap from the feature of different resolutions, which makes the final segmentation results of the decoder appear more burr edges. Compared with the segmentation method based on CNN, the segmentation models based on transformer and CNN, such as TransUNet and SwinUNet, have certain advantages. This is despite the fact that they combine the dual advantages of local modeling and long-range modeling. However, from the segmentation visualization results, there are still edge segmentation methods and under-segmentation and mis-segmentation parts. The reason is that the segmentation method based on transformer needs to experiment on a large amount of data to obtain ideal segmentation results. However, for the segmentation of medical image, due to the small number of data samples and the large difference in the structure of the data itself, the segmentation results are poor.

Compared with the based on CNN and transformer, the based on Mamba has great advantages. The model combines the advantages of CNN and transformer, and has low model parameters, fast, and less computer resources. From the segmentation results of VM-UNet and UVM-UNet, it can be seen that the lesion area in the prostate image can be effectively segmented, the segmentation results of VM-UNet and UVM-UNet are closer to those of mask, but there there are also instances of slight over and under-segmentation. The primary reason may be that VM-UNet and UVM-UNet overly focus on global dependencies, leading to diminished perception of local details. This compromises segmentation edge accuracy and sensitivity to small targets. As shown in the Fig 7, segmentation boundaries extend beyond the lesion area.

It can be seen from Fig 7 that the LM-UNet proposed in this paper has the best segmentation results. A novel PV-Mamba is introduced into the UNet, which has long modeling ability and low parameter quantity. At the same time, the encoder and decoder are constructed with CNN. In the construction process, the importance of the edge information of the lesion to the segmentation result is considered, so that the EFE is constructed between the shallowest and deepest layers of the encoder to extract the edge information at different resolutions in different encoders. Then the EFF is constructed to fuse the edge information with the output of each encoder to obtain the features with edge information in each encoder. To alleviate the semantic difference between the encoder and the decoder, MMSC is constructed between the encoder and the decoder for multi-stage and multi-level fusion, thereby improving the segmentation accuracy of the model. It can be seen that the LM-UNet segmentation model proposed in this paper has great application potential in prostate segmentation.

**Fig 8:**
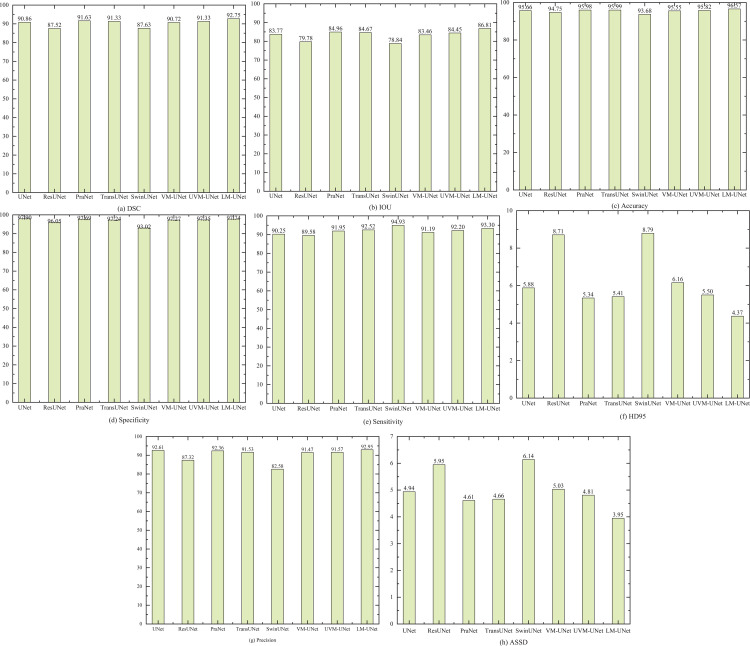
Bar Chart of Evaluation Metrics for Different Segmentation Methods.

The bar chart in Fig 8 clearly illustrates the segmentation performance of different segmentation methods. DSC, IoU, Accuracy, Specificity, Sensitivity, HD95, Precision, and ASSD are used as evaluation metrics. Among these, LM-UNet was evaluated as the overall best model, achieving improvements of 1.89, 3.04, 0.91, 3.05, and 0.34 in DSC, IoU, Accuracy, Sensitivity, and Precision respectively compared to the UNet, while reducing HD95, and ASSD metrics by 1.51, and 0.99, Specificity decreased by 0.16. Relative to SwinUNet, enhancements in DSC, IoU, Accuracy, Specificity, and Precision were noted at 5.12, 7.97, 2.89, 4.72, and 10.37, respectively, whereas HD95 and ASSD experienced declines of 4.42 and 2.19. Sensitivity, however, dipped by 1.63. In contrast to VM-UNet, improvements in DSC, IoU, Accuracy, Specificity, Sensitivity, and Precision by 2.03, 3.35, 1.02, 0.47, 2.11, and 1.48, respectively, while HD95 and ASSD saw reductions of 1.79 and 1.08. When juxtaposed with UVM-UNet, the achieved gains in DSC, IoU, Accuracy, Specificity, Sensitivity, and Precision by 1.42, 2.36, 0.75, 0.39, 1.10, and 1.35, respectively, and concurrently witnessed decreases in HD95, and ASSD by 1.13 and 0.86. Additionally, the standard deviation of each evaluation metric indicates that LM-UNet exhibits the lowest standard deviation, suggesting smaller data fluctuations, a more concentrated distribution, and higher consistency. This demonstrates that LM-UNet possesses superior stability.

### 3.5. Ablation study

The UVM-UNet is used as the baseline, and the LM-UNet components are mainly divided into EMA, EFE, EFF, and MMSC. To verify the improvement strategy of the segmentation algorithm in this paper, the influence of the above components on the performance of the segmentation algorithm is verified respectively. The experimental results are as Table 5 shows that the EMA is introduced into the encoder in the baseline network, and the segmentation effect of the model is improved compared with the baseline. It shows that the EMA has good performance by parallel processing feature aggregation through cross-spatial learning. When the EFE and the EFF are introduced respectively, the model segmentation performance is improved slightly, indicating that the PV-Mamba has certain advantages in long-distance modeling. When the EFE and the EFF simultaneously perform feature extraction and fusion, the segmentation performance of the model is greatly improved. Compared with baseline, DSC, IoU, Accuracy, Specificity, Sensitivity and Precision increased by 1.42, 2.36, 0.75, 0.39, 1.10, and 1.38, respectively, and HD95, ASSD decreased by 1.13, 0.86.

**Table 5:** Ablation experiments of different components.

Components				Metrics							
Baseline	EMA	EFE	EFF	DSC	IoU	Accuracy	Specificity	Sensitivity	HD95	Precision	ASSD
✓				91.33	84.45	95.82	97.35	92.20	5.50	91.57	4.81
				±5.45	±8.26	±1.72	±2.23	±6.86	±3.70	±9.22	±2.21
✓	✓			91.94	85.60	96.27	97.67	92.44	4.87	92.47	4.36
				±6.36	±8.93	±1.53	±2.04	±8.17	±3.82	±8.51	±2.08
✓	✓	✓		92.57	86.49	96.43	97.67	93.22	4.56	92.64	4.11
				±4.73	±7.42	±1.51	±1.87	±5.75	±3.45	±7.76	±1.89
✓	✓		✓	92.53	86.47	96.46	97.53	**93.58**	4.53	92.36	4.08
				±5.06	±8.00	±1.58	±2.07	**±6.68**	±3.62	±8.07	±2.09
✓	✓	✓	✓	**92.75**	**86.81**	**96.57**	**97.74**	93.30	**4.37**	**92.95**	**3.95**
				**±4.65**	**±7.44**	**±1.46**	**±1.87**	±6.29	**±3.28**	**±7.52**	**±1.86**

Compared with the baseline + EMA, DSC, IoU, Accuracy, Specificity, Sensitivity, and Precision increased by 0.81, 1.21, 0.30, 0.07, 0.86, and 0.48, respectively, and HD95, ASSD decreased by 0.50, 0.41. In addition, the standard deviation of the LM-UNet is mostly the lowest, indicating that the model has good stability.

### 3.6. Complexity analysis

**Fig 9:**
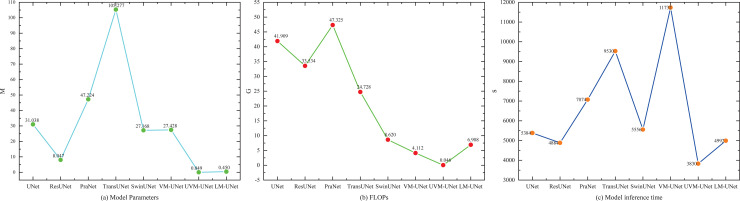
Complexity analysis of different methods.

To analyze the complexity of different segmentation methods, we conducted a quantitative analysis of their parameters, FLOPs, and inference time required for training 100 epochs, as shown in Table 6 and Fig 9. Fig 9 (a) demonstrated the number of parameters for different methods, with TransUNet having the most parameters at 105.277 M. The parameter of UVM-UNet is the lowest, only 0.049 M. The parameters of LM-UNet amount to 0.45 M, ranking second among all methods. Fig 9 (b) indicated the FLOPs of different methods, where PraNet has the highest FLOPs at 47.325 G. UVM-UNet has the lowest FLOPs at only 0.046 G. and LM-UNet has FLOPs of 6.908 G. Fig 9 (c) observed the inference time required for training different methods over 100 epochs. Among all methods, VM-UNet required the longest time, reaching 11,732 s. LM-UNet inference time was 4,992 s. Overall, while LM-UNet may not be the best in terms of parameters, FLOPs, and inference time. Compared to UVM-UNet, LM-UNet does exhibit certain differences in terms of parameters, FLOPs, and inference time. However, the EMA, EFE, and EFF components in LM-UNet effectively enhance boundary feature extraction capabilities while simultaneously integrating features from different levels. A comprehensive analysis of its segmentation results reveals that this method achieves an outstanding balance between segmentation quality and computational complexity. A good balance between segmentation quality and computational complexity.

**Table 6:** Complexity analysis of different methods.

Method	Parameters (M)	FLOPs (G)	Inference Time (s)
UNet	31.038	41.909	5,384
ResUNet	8.047	33.534	4,884
PraNet	47.224	47.325	7,074
TransUNet	105.277	24.728	9,530
SwinUNet	27.168	8.620	5,556
VM-UNet	27.428	4.112	11,732
UVM-UNet	0.049	0.046	3,830
LM-UNet	0.527	6.908	4,992

## 4. Conclusion

Aiming at the characteristics of fuzzy boundary and low contrast of lesions in prostate MRI images, the traditional UNet segmentation network can not effectively extract edge information, as well as the loss of edge detail information after multiple downsampling, and the problem of feature fusion of different resolutions in encoder and decoder. We propose a LM-UNet prostate MRI image segmentation method. This method introduce the PV-Mamba in the deep layer of the encoder-decoder. PV-Mamba, as a linear state selection model, not only reduces the number of model parameters but also exhibits strong long-range modeling capabilities. We construct EFE between the shallowest and deepest layers of the encoder to effectively extract edge detail information of lesions at different encoder levels. Meanwhile, we build EFF to fuse the extracted edge detail information with the output results of each encoder. Additionally, utilizing MMSC to achieve multi-level and multi-scale overlapping between encoders and decoders, contextual semantic discrepancies are reduced, resulting in smoother segmentation outcomes. The experimental results show that the proposed LM-UNet not only achieves higher segmentation accuracy, but also has a smaller parameter size and lower computational memory.

This study has several limitations: (1) The experiment was conducted solely on a single dataset, failing to adequately reflect the complexity and diversity of medical imaging data encountered clinical practice. (2) The research primarily focused on segmentation performance while paying less attention to real-time processing capabilities. In clinical, algorithm execution speed significantly impacts physicians diagnostic and treatment decisions. (3) The experiment utilized only 2D slices, disregarding 3D contextual information. Our future work will expand both the validation dataset and the scope of research.
